# *In-Vitro* Study of the Effect of Anti-Hypertensive Drugs on Placental Hormones and Angiogenic Proteins Synthesis in Pre-Eclampsia

**DOI:** 10.1371/journal.pone.0107644

**Published:** 2014-09-24

**Authors:** Subrata Gangooly, Shanthi Muttukrishna, Eric Jauniaux

**Affiliations:** 1 Institute for Women’s Health, University College London, London, United Kingdom; 2 Anu Research Centre, Department of Obstetrics and Gynaecology, University College Cork, Cork University Maternity Hospital, Cork, Republic of Ireland; Medical University Innsbruck, Austria

## Abstract

**Introduction:**

Antihypertensive drugs lower the maternal blood pressure in pre-eclampsia (PE) by direct or central vasodilatory mechanisms but little is known about the direct effects of these drugs on placental functions.

**Objective:**

The aim of our study is to evaluate the effect of labetolol, hydralazine, α-methyldopa and pravastatin on the synthesis of placental hormonal and angiogenic proteins know to be altered in PE.

**Design:**

Placental villous explants from late onset PE (n = 3) and normotensive controls (n = 6) were cultured for 3 days at 10 and 20% oxygen (O_2_) with variable doses anti-hypertensive drugs. The levels of activin A, inhibin A, human Chorionic Gonadotrophin (hCG), soluble fms-like tyrosine kinase-1 (sFlt-1) and soluble endoglin (sEng) were measured in explant culture media on day 1, 2 and 3 using standard immunoassays. Data at day 1 and day 3 were compared.

**Results:**

Spontaneous secretion of sEndoglin and sFlt-1 were higher (p<0.05) in villous explants from PE pregnancies compared to controls. There was a significant time dependant decrease in the secretion of sFlt-1 and sEndoglin in PE cases, which was seen only for sFlt-1 in controls. In both PE cases and controls the placental protein secretions were not affected by varying doses of anti-hypertensive drugs or the different O_2_ concentration cultures, except for Activin, A which was significantly (p<0.05) higher in controls at 10% O_2_.

**Interpretation:**

Our findings suggest that the changes previously observed in maternal serum hormones and angiogenic proteins level after anti-hypertensive treatment in PE could be due to a systemic effect of the drugs on maternal blood pressure and circulation rather than a direct effect of these drugs on placental biosynthesis and/or secretion.

## Introduction

Normal placentation in human pregnancy is characterized by deep invasion of the placental bed by the extravillous trophoblast through the decidua down to the inner or junctional zone of the uterine myometrium [1]. Placental-related diseases of pregnancy are almost unique to the human species and affect around a third of human pregnancies [2]. These diseases include mainly miscarriages and pre-eclampsia (PE), which are respectively at the opposite end of a spectrum of major disorders of the development of the utero-placental interface. In miscarriage, placentation is severely impaired from an early stage leading to complete degeneration and rapid collapse of the placental structure before the end of the first trimester [2]. In PE, placentation is sufficient to allow partial development of the placenta but too shallow for complete development of the utero-placental circulation and normal fetal growth during the second half of pregnancy [3], [4].

The incomplete conversion of the end branches of the uterine circulation in PE results in retention of smooth muscle cells within their walls [5], [6]. As a consequence, some vaso-reactivity persists in the utero-placental vascular bed leading not only to reduced perfusion of the intervillous chamber of the definitive placenta but also to intermittent perfusion exposing the placental tissue to low grade ischemia-reperfusion and chronic oxidative stress [7], [8]. Chronic oxidative stress inside the placenta leads to progressive damage of the villous tissue, fetal growth restriction and finally to diffuse maternal endothelial cell dysfunction and clinical PE [2], [6], [9], [10].

The exact pathogenic molecular mechanisms leading to the systemic endothelial dysfunction of PE remain to be determined [11]. The endothelial dysfunction associated with PE involves multiple maternal organ systems including the placental tissue itself resulting in major functional changes and progressive “placental insufficiency” [12]. Intra-placental oxygen (O_2_) distribution is likely to be an important regulator of trophoblast function in PE [6], [10] but other factors such as immune maladaption, excessive shedding of trophoblast debris, oxidative stress and genetic factors have been found to contribute to the pathogenesis of the abnormal placentation [2], [11], [13]. Production of placental anti-angiogenic factors and in particular, soluble fms-like tyrosine kinase-1 (sFlt-1) and soluble endoglin (sEng), have been shown to be up-regulated in PE and their levels are found to be increased in maternal circulation, weeks before the onset of the disease [14], [15]. Placental anti-angiogenic factors are released into the maternal circulation and are known to disrupt the maternal endothelium functions resulting in hypertension, proteinuria and the other systemic manifestations of PE [11], [12].

Anti-platelet agents, alpha- and beta-blockers, calcium channel blockers, diuretics vasodilators (NO agents) and magnesium sulphate are the main drugs used in the management of PE [11]. Recent research using animal models to evaluate the different secondary effects of the disease has revealed some of the underlying mechanisms of PE [4]. However, PE is a disorder of deep invasive placentation, limiting the study of pharmacologic interventions to humans and a few other higher primates. We have previously shown the anti-hypertensive drug α-methyldopa (Mdopa) has an effect on maternal serum levels of angiogenic proteins and placental hormones in pregnancies complicated by PE [16], [17]. This effect may be independent of Mdopa known antihypertensive central action and we have suggested that Mdopa may directly influence trophoblastic protein synthesis and/or release and thus some of the main placental biological functions. The aim of this study was to further investigate the possible effect of Mdopa and the other drugs, routinely used in the management and prevention of PE, on the placental secretion of hormones and angiogenic protein known to be altered in pregnancies complicated by PE.

## Materials and Methods

Women booked for elective delivery by caesarean section at term at University College London Hospital (UCLH) were recruited for this study. Women with a multiple pregnancy, with a history of smoking, assisted reproductive treatment or with any pre-existing medical disorders such as chronic hypertension, diabetes, renal disease or immune disorders were excluded from the study.

In the PE group (n = 3), the diagnosis of late onset PE was confirmed using clinical and biochemical parameters [18]. The maternal blood pressure (BP) was measured in duplicate using a standard mercury sphygmomanometer and the average of two readings taken. Korotkoff sounds 1 and 5 were used to define systolic and diastolic BP respectively. The mean BP was calculated as diastolic BP+1/3 pulse pressure. PE was defined according to the guidelines of the International Society for the Study of Hypertension in Pregnancy [19]. The diagnosis of PE was based on two recordings of diastolic blood pressure 90 mm Hg, at least four hours apart; or one recording of diastolic BP≥120 mm Hg, in a previously normotensive woman; and urine protein excretion ≥300 mg in 24 hours, or two readings of ++ or more on dipstick analysis of a midstream or catheter specimen of urine, if no 24 hour collection was available. In all 3 cases, the average resistance and pulsatility indices were above the 95^th^ centile of the normal ranges.

The control group (n = 6) included only uncomplicated singleton spontaneous conceptions booked for elective caesarean section at term. The indication for caesarean section in the controls was a previous caesarean section in five cases and a fibroid in lower segment with fetal transverse presentation in one case.

The study was approved by the Joint UCL/UCLH Committees on the Ethics of Human Research (Reference Number: 05/Q0505/82). All women received information about the study and written consent was obtained prior to the caesarean section.

### Samples

In both groups the placenta was collected immediately from the operating theatre during a caesarean section. The placenta was kept on a sterile tray and sterile forceps and scissors were used to obtain five placental samples from central part. The samples were then rinsed thoroughly between 5 to 7 times with sterile HBSS (Hanks balanced salt solution, GIBCO, UK) to remove all blood and blood clots. The villous samples were immediately transferred to the laboratory, placed under the laminar flow hood and cut into smaller placental biopsies of maximum 1 cm in diameter. Each villous biopsy was weighed in a sterile Bijou bottle (Sterilin Ltd, Staffordshire, UK) before transfer to the culture well containing the medium.

### *In vitro* cultures

Villous samples were cultured in sterile 12 well culture plates (Nunc-immunoplate, SIGMA-Aldrich Co, UK). The culture media contained Dulbecco’s modified eagle medium (DMEM, GIBCO, UK) with glutamate and 10% fetal calf serum (SIGMA-Aldrich Co, UK); 100 U/ml penicillin and 100 µg/ml streptomycin. Each culture well-contained 1.5 ml of culture media and were cultured in a humidified 5% CO_2_ incubator at 37°C, for 3 days.

We tested the following drugs at 3 different dosages as previously described by Xu et al., [20], [21]: labetolol (LABE) (low dose 15.6 µgm/ml; medium dose 250 µg/ml; high dose 4000 µg/ml), hydralazine (HDZ) (low dose 5 µg/ml; medium dose 125 µg/ml; high dose 1000 µg/ml;), Mdopa (low dose 39 µg/ml; medium dose 625 µg/ml; high dose 10000 µg/ml) and pravastatin sodium (low dose 0.039 µg/ml; medium dose 1.25 µg/ml; high dose 20 µg/ml). Control medium included 75 µl of HBSS (GIBCO, UK) for LABE, HDZ and pravastatin and 75 µl of 0.05 M of HCL for Mdopa.

For each experiment, two sets of culture plates/drug from both groups were incubated in 2 different CO_2_ incubators at ambient atmospheric O_2_ concentration (20%) and 10% O_2_ concentration as previously described [5].

After 24 hours of incubation (day 1), the culture supernatant from all culture experiment was collected and frozen at −80 c until assayed. The villous explants were washed three times with HBSS (GIBCO, UK) and 1.5 ml of fresh culture medium and 75 µl of the different drug concentration solutions was added. The same procedure was repeated on day 2 and day 3. For each sample, after 24 h and 48 h, the media was removed and the villous explants were washed and replaced with fresh culture media and drug solutions.

All our experiments were carried out in triplicate wells and minimum repeated 3 times totalling 540 samples analyzed for the entire experiment.

### Immunoassays

Total activin A (follistatin bound and unbound) was measured using a two-site enzyme linked immunosorbent assays ELISA [22], [23]. Affinity purified human activin A was used as the assay standard. The detection limit of the assay for purified human Activin A was 50 ng/ml. Intra- and inter assay coefficients of variation were 9% and 10%, respectively.

Dimeric inhibin A was measured using a two-site ELISA [25], [26]. The sensitivity of the assay was 2 pg/ml and the intra- and inter- assay variations were 5.2% and 6.5%, respectively.

hCG was measured using a commercially available kit from IBL with standards at concentrations of 0 miu/ml, 5 miu/ml, 25 miu/ml, 100 miu/ml, 200 miu/ml.

Human sFlt-1 and sEng were measured using a two-site (ELISAs) from R & D Systems (Minneapolis, Minnesota, USA). Measurements were conducted in duplicate according to the manufacturer’s protocol. The assay was validated in our laboratory and dilution curves created to get the correct dilution for each assay. The minimum detectable levels for sFlt-1, and sEng were 5 pg/ml and 7 pg/ml respectively. Intra- and inter-assay coefficients of variation respectively in our laboratory were as follows: sFlt-1 7%, 9%; and sEng 6%, 8%, as previously described [17].

All measured concentrations were normalised against the gram (g) weight of the tissue sample.

### Statistical Analysis

The data were analyzed using the Pad Prism 5 statistical data analysis and statistical software package (GraphPad Software, La Jolla, CA, USA). Standard Kurtosis analysis indicated that some values were not normally distributed and the results are presented as median and standard deviation (SD). A non-parametric analysis Mann-Whitney (Wilcoxon) W test was used to compared cases and controls. A Kruskal-Wallis test with Dunns post hoc tests was performed to evaluate statistical differences when more than 2 variables were tested. A P value of <0.05 was considered statistically significant.

## Results

There was no significant difference in the median maternal age, parity, body mass index (BMI) and gestation at delivery between the PE and controls. The systolic and diastolic BP, levels of proteinuria and of all hepatic enzymes were significantly (P<0.001) higher in the PE compared to controls.

### Baseline secretion

**Figure 1:**
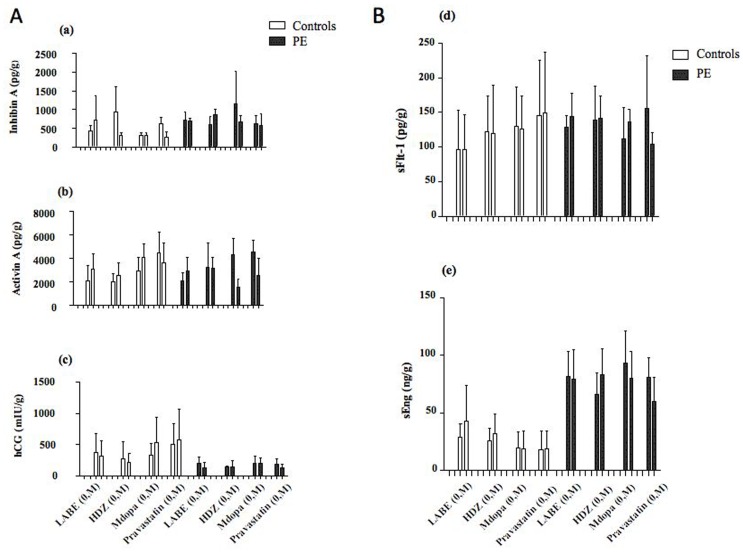
Comparison of the effect of no drug (0) compared with maximum (M) dose for LABE (4000 mg/ml), HDZ (1000 mg/ml), Mdopa (10,000 mg/ml) and Pravastatin (20 mg/ml) after 1 day in culture at ambient O_2_ on the median (SD) levels of placental hormones (Fig. 1A) and angiogenic protein (Fig. 1B). The beta errors for no drug versus max dose of Mdopa are: 0.0465 & 02451 on Inhibin A; 0.0001 & 0.9761 on Activin A; 0.0019 & 0.0367 for hCG; 0.0655 & 0.0054 for sFlt-1 and 0.0559 & 0.1977 for sEng, respectively.

After 1 day in culture at ambient O_2_ (20%), the median levels of sFlt-1 and sEng were significantly (P<0.05) higher in villous explants from pregnancies presenting with PE than in normal controls (figure 1). There was no difference in the levels of activin A, inhibin A and hCG after 1 day in culture between the PE and control group.

### Effect of Time

There was a significant (P<0.001) decrease in all protein median levels over the 3 days in culture at ambient O_2_ in both PE samples and controls (figure 1). The most pronounced changes were found in PE explants, which showed a 10-fold decrease for Sflt1 and a 3-fold decrease for sEng, between day 1 and day 3 in culture.

### Effect of O_2_ concentrations

There was no difference in the placental hormones and angiogenic protein secretion at different O_2_ concentrations, except for the secretion of activin A which was significantly (P<0.05) increased in the controls at 10% O_2_ during the first day in culture and second day. This effect was not observed after 3 days in culture.

### Dose dependant effect of anti-hypertensive drugs

No significant difference was found in villous explants secretion in both controls and PE cases when cultured for up to 3 days with no drug (control media zero concentration) compared with increasing doses of LABE (15.6, 250 and 4000 µg/ml), HDZ (5, 125 and 1000 µg/ml), Mdopa (39, 625 and 10,000 µg/ml) or Pravastatin (0.039, 1.25 and 20 µg/ml) at ambient or 10% O_2_ concentration. Figures 1A and 1B illustrates the effect of no drug compared with maximum dose for each drug tested after 1 day in culture at ambient O_2_ on the median levels of placental hormones and angiogenic protein.

## Discussion

Our study shows that in vitro secretion of angiogenic proteins sEng and sFlt-1 is higher in villous tissue obtained at the time of elective caesarean section performed at term in women presenting with late PE compared to normal controls obtained under the same experimental condition. By contrast, in vitro secretion of placental hormones inhibin A, activin A and hCG is not different in PE than in controls although these proteins have been found in higher concentrations in the circulation of women with PE, months before they present with the clinical symptoms of the disease. In both cases and controls, the secretion of all proteins tested was time-dependent in vitro but not affected by the addition of anti-hypertensive drugs or statins to the culture medium.

Tissue culture experiments have been previously used In PE to evaluate the synthesis of various proteins and to test for the effect of different O_2_ concentrations [24], [25]. We found only two previous studies on the effect of clonidine, diazoxide, frusemide and hydralazine (HDZ) on placental production of sFlt-1, sEng cytokines over 24 hours in placental explants from term pregnancies complicated by PE [20], [21]. With the exception of frusemide, none of the other drugs tested in culture over 24 hours had any effect on sFlt-1 and sEng production in placental explants from both PE and normal controls. Furthermore, except for HDZ, the other drugs studied are now rarely used in clinical practice for the treatment of PE and other hypertensive disorders of pregnancy [26], [27]. Intravenous HDZ or LABE are both considered first-line drugs for the management of acute and severe hypertension during pregnancy whereas oral Mdopa or LABE are used most commonly in the management of non-severe hypertension [27].

The pharmacokinetic, pharmacological effects and pharmacogenetic of these drugs are very different. HDZ acts by dilating resistance arterioles, thus reducing peripheral resistance and has a direct catecholamine-mediated positive inotropic and chronotropic stimulation of the heart, resulting in an increase in cardiac output [28]. Mdopa is a DOPA analogue, which is converted to α methyl-norepinephrine an agonist of presynaptic central nervous system α2-adrenergic receptors [29]. Activation of these receptors inhibits sympathetic nervous system output and lowers blood pressure [30]. LABE is a β-blockers with antagonistic properties at both α- and β-adrenergic receptors, with direct vasodilator activity [31]. HDZ appears to activate guanylatecyclase, leading to increase cyclic GMP in arterial vascular smooth muscle and causing vasorelaxation [32]. The data of the present study indicate that the anti-hypertensive drugs do not modify the trophoblastic secretions of placental hormones and angiogenic proteins and up to 3 days in culture suggesting that these drugs do not have a direct effect on placental biosynthesis.

Clinical PE is associated with deficient intravascular production of prostacyclin and excessive production of thromboxane, which is a vasoconstrictor and stimulant of platelet aggregation. This observation has led to the concept two decades ago that anti-platelet agents, mainly aspirin can be used from early in pregnancy to prevent the development of PE [33]. 3-Hydroxy-3-methylglutaryl-coenzyme A (HMG-CoA) reductase is the key enzyme of cholesterol synthesis and statins which are HMG-CoA reductase inhibitors have been increasingly used in patients with hypercholesterolemia and/or at risk of vascular diseases [34]. Pravastatin and other statins have also been shown to reverse various pathophysiologic pathways associated with PE, such as angiogenic imbalance, endothelial injury, inflammation, and oxidative stress [35]. Pravastatin has been shown to induce the VEGF-like angiogenic factor placental growth factor (PGF) in a mouse model but there are no data on its possible pharmacological effects on the human placenta. The results of the present study indicate that similarly to anti-hypertensive drugs, pravastatin does not have a direct effect on placental biosynthesis of placental hormones and angiogenic proteins.

Inhibin A, activin A and hCG are all produced by the villous trophoblast and known to be increased in the second trimester serum samples from women presenting with clinical PE later in pregnancy [36]. There is mounting evidence that oxidative stress or an imbalance in the oxidant/antioxidant activity in utero-placental tissues plays a pivotal role in the development of PE [2]. Uterine contractions during labor are known to be associated with intermittent utero-placental perfusion and placentas subjected to prolonged active labor shown biological changes similar to those observed in PE [37]. This is further demonstrated by the increase in activin A and sFlt-1 maternal serum levels during labor compared to pre-labor in PE [36]. We have previously shown that the first trimester human placenta syncytiotrophoblast is acutely sensitive to O_2_-mediated damage [38] and that the changes in intra-placental O_2_ concentration during the first half of pregnancy are essential for trophoblast differentiation and placental development [39], [40]. Furthermore, we recently found a direct relationship in the early intrauterine PaO_2_ in vivo and inhibin A and sFLT-1 concentrations in placental bed blood confirming our hypothesis by intrauterine O_2_ tension play a role in the placental proteins synthesis [41]. It has also been recently suggested that the oxidative status of the trophoblast may regulate glycosylation of proteins and thereby modulate major trophoblast cell functions [42]. In the present study, we found a significant increase in-vitro of activin A synthesis during the first two days in culture at 10% O_2_ in normal controls suggesting that intra-placental O_2_ tension may have an effect on some placental synthesis during all three trimesters in normal pregnancy. However, compared to the first trimester trophoblast, which develops in 8–10 O_2_ [2], [5], the third trimester villous tissue biological activities are less likely to affected by changes in O_2_ tension in culture.

Activin A is know to promote the invasion of first-trimester cytotrophoblast until 10 weeks gestation [43] may play a role in the pathogenesis of PE by inducing excessive apoptosis in placenta indirectly through enhancing Nodal expression [44], [45]. It has been recently suggested that the increase in placental secretion of inhibin A, activin A and hCG results from premature accelerated differentiation of the villous cytotrophoblasts and could be linked to chronic intra-placental oxidative stress.

PE is almost exclusively a disorder of human deep placentation, in-vitro cultures of human placental explants are essential to better understand the pathophysiology of PE but also the impact of anti-hypertensive drugs on placental biology. As shown in the present study, the biosynthesis of these placental hormones does not seem to be influenced by the presence of anti-hypertensive drugs or Pravastatin in the culture medium. Our study is limited to late-onset PE and included only three cases but we assume that the samples represent the same process and are therefore justified in repetitive sampling of three placenta specimens. The effects of anti-hypertensive drugs are not restricted to the placenta but a better understanding the impact of these drugs on placental functions in PE is essential to the development of more selectively targeted drugs ensuring swift introduction of optimal treatment whilst minimizing the use of inappropriate or ineffective drugs.
